# Supplementary material for: LRFMV: An efficient customer segmentation model for superstores
Source: PLoS One. 2022 Dec 20;17(12):e0279262. doi: 10.1371/journal.pone.0279262 (PMC9767363; doi:10.1371/journal.pone.0279262)
Supplement: Attachment — Submitted filename: Response to Reviewers.pdf [file pone.0279262.s001.pdf]

Original Manuscript ID: PONE-D-21-32764R1

Original Article Title: “LRFMV: An Efficient Customer Segmentation Model for Superstores”

To: The Editor in Chief, PLOS ONE

Re: Response to reviewers

Dear Sir,

Thank you for enabling us to resubmit our paper so that we may respond to the reviewers' remarks. We would like to express our deepest gratitude and appreciation for the feedback, which really assisted us in improving the paper's standard. All of the suggestions were carefully considered, and the manuscript was revised. When replying to a comment, we initially give a broad overview of the problem before mentioning the essential changes made in the updated document. We also updated the methods section of our manuscript and mentioned that the customer names and other identifying characteristics in the dataset used are fictional. The comprehensive responses and explanations to the reviewers' comments and suggestions can be found below.

We have uploaded (a) a point-by-point response to the reviewers' suggestions (answer to reviewers), (b) an updated manuscript with yellow highlighting showing changes, and (c) a clean, updated version without highlights (reaction to reviewers) (PDF main document).

We have responded to the comments of Reviewer 1 and Reviewer 2 in this reply letter in the order in which they were made.

Thank you for your consideration.

Best regards,

Dr. Md. Golam Rabiul Alam

(Corresponding Author)

RESPONSE TO REVIEWER #1'S COMMENTS

Reviewer#1, Concern # 1: The data obtained from the LRMFV model is clustered by using conventional K-means, K-Medoids, and Mini Batch K-means methods. Provide some rationalities why these models have been chosen for this clustering?

Author response: We would like to express our deepest gratitude to the honorable reviewer for the insightful comment and we are much obliged for the valuable suggestions. Many clustering methods are used for grouping similar data objects inside the same set based on similarity criteria, such as density-based, centroid-based, and hierarchical clustering. Density-based algorithms link core objects and their surroundings to generate dense regions known as clusters, which are asymmetrical and made up of the most densely connected points. Furthermore, the hierarchical clustering method merges or separates related data objects by creating a hierarchy of clusters, also known as a dendrogram, where the same objects can reside in multiple clusters. By hypothesizing a model for each cluster, model-based clustering techniques discover the best fit of data for a particular model. Grid-based algorithms use dense grids to generate clusters and use multi-resolution grid data structures. It quantizes the original data space into a finite number of cells that constitute the grid structure before performing all operations on the quantized space. However, the key objective of this research is to partition the customers based on L, R, F, M, and V without overlapping the cluster elements. Therefore, we have chosen partition-based clustering methods i.e., K-Means, Mini-batch K-Means, and K-Medoids for the customer segmentation.

For the large and diversified data set like our superstore dataset, centroid-based clustering like K-Means was the preferred one as it is fastest and scalable. The Mini-batch K-Means can reduce the time it takes to compute a growing dataset. However, K-Means and Mini-batch K-Means algorithms are sensitive to outliers. The K-Medoid mitigates sensitivity to outliers whilst still ensuring convergence.

Author action: Updated sections have been marked yellow using the highlighter tool in the revised

manuscript.

Modifications made to the manuscript in this regard is as follows:

[Updated] [Section: 1.1 Background and Motivation]

A partitioning or clustering method begins by creating an initial set of k partitions, where k is the number of partitions to construct. It then employs an iterative relocation strategy, in which objects are moved from one group to another in an attempt to enhance partitioning. The criterion that distinguishes partition-based algorithms is that objects in the same cluster are close or related to each other, whereas objects in other clusters are far apart and unrelated and this makes partition-based algorithms different and more popular from other clustering algorithms. The data points are partitioned into one level with some clustering approaches. K-Means, Mini-batch, and K-Medoid are examples of such techniques.

Despite the presence of a large range of clustering algorithms, k-means, k-medoids, and mini-batch were chosen for this research work after examining some key functionalities that are closely related to the dataset in this concern. K-means algorithm is known for its speedy execution time and scalability. The Mini Batch K-Means algorithm is a reliable and efficient method for clustering with similar characteristics while lowering the costs of processing such massive amounts of data. In the case of K-medoids, instead of using the centroid of the items in a cluster as a reference point as k-means clustering does, clustering uses the medoid as a reference point. A medoid is an object in the cluster that is most centrally placed or has the least average dissimilarity to all other objects which makes K-medoids more noise-resistant. Moreover, these three algorithms ensure that the same data points do not belong to multiple clusters. Since they do hardly allow repetitive data points in different clusters, these partitioning techniques are relatively redundant in terms of cluster nodes.

In the case of K-means clustering, the algorithm has successfully completed the clustering procedure or grouping of data points in k number of clusters for the given dataset. If the centroids (k values) in k-means remain at the same position or in point for two iterations, the algorithm ensures that it has completely grouped the data points into correct clusters. It can simply be adapted to new data sets and new examples can be generated. It can be applied to clusters of all forms and sizes, including elliptical clusters.

K-Medoids clustering is a partition-based technique that includes the ability to generate empty clusters and has the sensitivity to outliers or noise. It also ensures convergence, identical to the k-means approach as well as determining the cluster's member with the most coherence.

Finding a cluster is made easier and cuts computational costs with the Mini-batch K-means technique. The mini-batch strategy was one of the chosen approaches for this paper since the dataset was large enough and will continue to grow in the future for this specific business market.

One of the goals of this study is to compare several algorithms in order to achieve the best possible result, and these three algorithms demonstrated their efficacy for our target dataset and business. We found closely related outputs when we compared the findings of K-Means, Mini-batch, and K-Medoid for our dataset, ensuring clustering accuracy.

Reviewer#1, Concern # 2: Please use labels for Figures 7-9

Author response: We would like to offer our sincere regrets for overlooking these issues and express our deepest gratitude to the honorable reviewer for specifying these through insightful comments. As the respected reviewer indicated, we have labeled the axes of Figures 7-9 in the updated manuscript. However, as an extra image (Figure 7) has been added in the updated manuscript, the Figures 7, Figure 8 and Figure 9 in the previous paper have been termed as Figure 8, Figure 9 and Figure 10.

Author action: Updated axes' labels of figures have been marked yellow using the highlighter tool in the revised manuscript.

Modifications made to the manuscript in this regard is as follows:

[Updated] [Section: 4.1 Comparative Analysis]

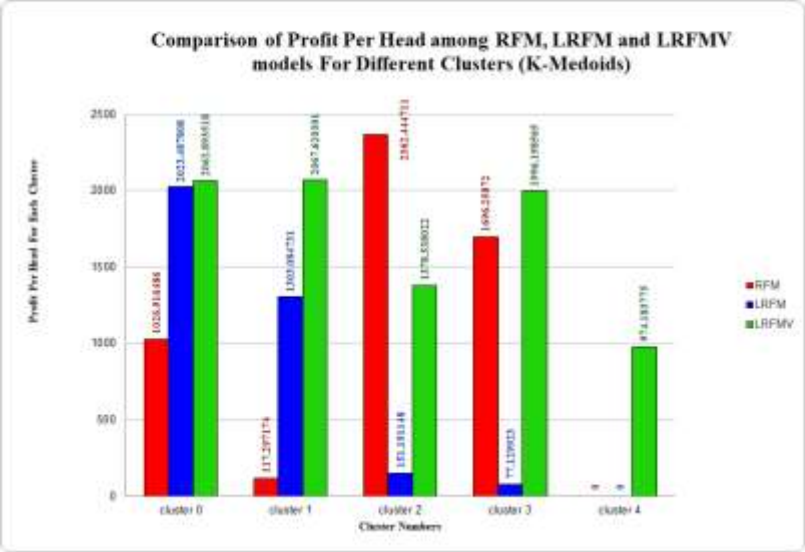

Figure 8. Profit analysis for RFM, LRFM and LRFMV models using K-Medoids algorithm

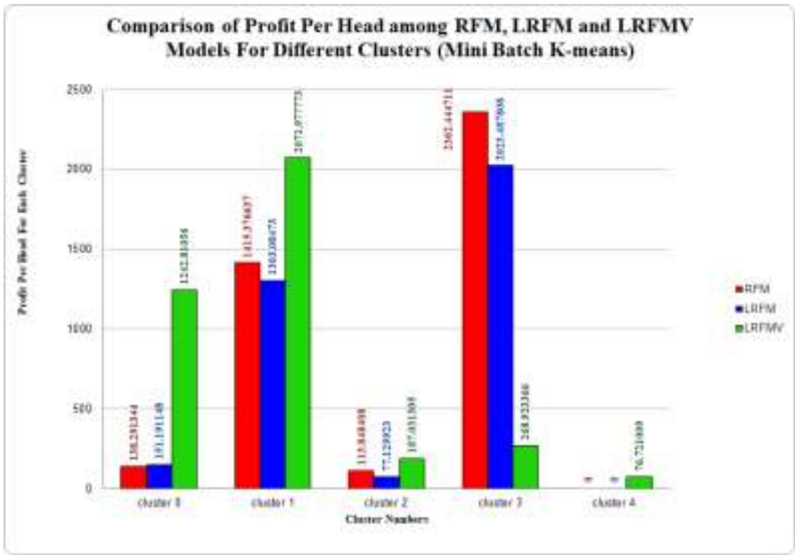

Figure 9. Profit analysis for RFM, LRFM and LRFMV model using Mini Batch K-means algorithm

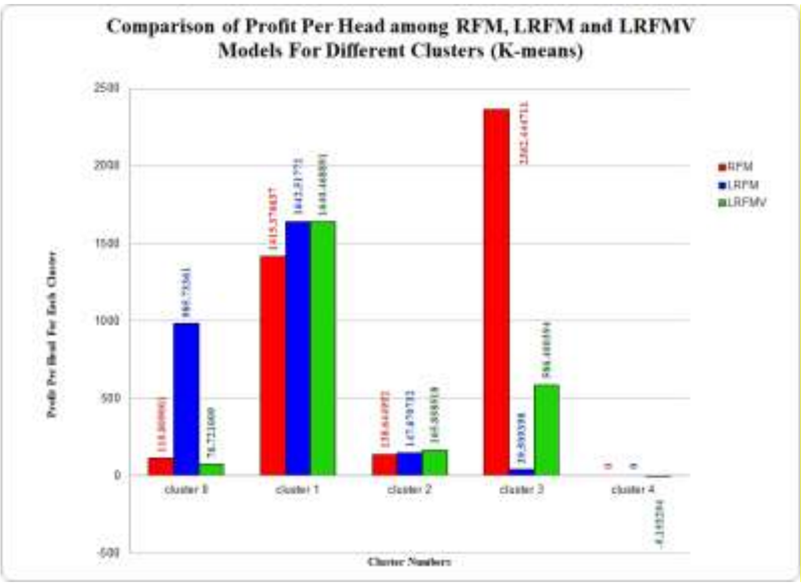

Figure 10. Profit analysis for RFM, LRFM and LRFMV models using standard K-means algorithm

Reviewer#1, Concern # 3: Please provide a paragraph about the technical contributions of this research study. Section 1.2 seems to summarize the benefits of the proposed method.

Author response: We would like to convey our heartfelt gratitude to the esteemed reviewer for his insightful comment. As recommended by the respected reviewer, we have added a paragraph about the technical contributions of this research in the introduction section of the revised manuscript.

Author action: Updated sections have been marked yellow using the highlighter tool in the revised manuscript.

Modifications made to the manuscript in this regard is as follows:

[Updated] [Section: 1.2 Contributions]

The proposed methodology demonstrates a novel technique for using unsupervised machine learning to develop a commercial solution. The key contributions of this research are the presentation of the correlation between the volume of products and the profit earned against each customer along with the findings from the evaluation of LRFMV model after applying the standard K-means, K-Medoids and Mini Batch K-means algorithms. Moreover, it compares the result of LRFMV analysis with traditional RFM and LRFM analysis. The following summarizes the major contributions of this research.

- This research introduces a new dimension V (volume) to the existing LRFM model in order to show a direct profit-quantity relationship in customer segmentation. The suggested method found a high connection between profits per head and the commodity bought by each client in a single transaction. The LRFMV model is capable of addressing a number of problems connected to identifying the optimum client for the optimal product.

- The proposed method has a unique blend of data preprocessing, scoring system, segmentation, and concluding results from the segments using a matrix. At first, some new features on their purchasing habits were analyzed using existing features where the existing features themselves were not picked for segmenting the customers in this case. The data was then reduced into five main features Length (3.3.1), Recency (3.3.2), Frequency (3.3.3), Monetary (3.3.4), and Volume (3.3.5) with their individual equations, which are identical to the scoring system. After that, the dataset was segmented based on the score.

- A comparison of different types of centroid-based clustering algorithms is also shown based on this model. Some famous clustering algorithms are K-means, Mini-Batch, and K-medoids, which belong to the same type but they work in different mechanisms. K-means minimize total squared error, whereas k-medoids tend to minimize the sum of dissimilarities between points. Mini-batch works like the K-Means algorithm with the fixed size of chunks. The differences in mechanism between these three algorithms also affect the end result of our model, which is clearly mentioned.

- Finally, a customer classification matrix was used in the suggested system to analyze the results with profit margin, revenue, and cost to serve for more insights.

RESPONSE TO REVIEWER #2'S COMMENTS

Reviewer#2, Concern # 1: The Author Summary can be improved.

Author response: We would like to express our deepest gratitude to the honorable reviewer for the insightful comment and we are much obliged for the valuable suggestions. Having said that, we have revised the Author Summary and updated it.

Author action: Updated sections have been marked yellow using the highlighter tool in the revised

manuscript.

Modifications made to the manuscript in this regard is as follows:

[Updated] [Author Summary]

Author Summary

Why was this study done?

- Superstore business has been booming in the last decades. In the FY-2017, retail revenue of the top 250 superstores is 4,530,059 million USD which achieved 5.7% economic growth [1].

- The Length, Recency, Frequency, and Monetary model, also known as the LRFM model, was introduced as an improved version of the RFM model to identify more relevant and exact consumer groups for profit maximization. However, there exists a substantial association between the purchase quantity and revenue generation that had been overlooked in earlier models. In this research, we introduced the LRFMV model, an improved version of existing business models for superstores, to further assess how much revenue boost and marketing strategy can be developed for the superstore industry and contribute to both technical sectors and the business world.

- In this research, we searched for a new way to utilize the segmentation model based on the scoring procedure and encountered how a business based matrix can employ them to have a substantial influence on the existing collaborative business and technology sector.

What did the researchers do and find?

- We proposed an efficient customer segmentation model, LRFMV and tried to observe the profit-quantity relationship. A new dimension V (volume) has been added to the existing LRFM model in order to show a direct profit-quantity relationship. Here, the V stands for volume, which was

derived by calculating the average number of products purchased by a frequent superstore client in a single day.

- To get the final average as volume in a specified time frame, the previously found average amount was divided by total days in a limited period of time of visitation of that customer. Quantity of purchased goods refers to the average amount of procured product by repeatedly going customers.
- Superstores have a varying number of different products with the record of being bought in different quantities multiple times on the same day by a specific customer. In RFM and LRFM models, the relationship between profit and purchased quantity and how they can contribute to an effective customer behavioral analysis was not investigated and evaluated.

What do these findings mean?

- It is visible that a large volume of purchased products is positively influencing the profit maximization of a superstore.
- The establishment of the proposed model will assist superstores in generating more profit and performing comprehensive business analysis by helping to find the most profitable group of customers.

Reviewer#2, Concern # 2: It is unclear what are significant benefits of incorporating volume in existing RFM or LRFM models.

Author response: Thank you for the astute comment and recommendation. We have incorporated how adding volume to existing RFM or LRFM models can be beneficial in both the technical and business worlds in the revised manuscript.

Author action: Updated sections have been marked yellow using the highlighter tool in the revised

manuscript.

Modifications made to the manuscript in this regard is as follows:

[Updated] [Section 3.3.5: Computation of V]

In today's world, it has become crucial to combine technical innovation and knowledge with business in order to create and explore new business opportunities. With this in mind, this paper attempted to establish a new technological component that would allow a company to enhance its profitability. With the existing LRFM model, a new feature V has been added with the goal of identifying the most profitable cluster of consumers that will ensure and offer a higher profit to a business. It can be used in a variety of industries to locate potential clients by analyzing their purchasing habits or tendencies.

Volume is a rescaled version of the number of goods purchased by a potential customer. It identifies a group of valuable customers for any company by highlighting the amount of their purchased product over a set period of time or a set number of visits. The proposed term by us in this research paper adds value to the LRFM model by figuring out the customer clusters that give more profit to an organization. It shows that if a customer buys a large amount of product in his certain visits regardless of the spent money, he will contribute more to the profit of that organization.

The higher the number of useful features in a business model, the higher the amount of information can be extracted from a dataset. Hence, we can have more freedom and flexibility in terms of choosing features for clustering. However, we must be cautious when selecting characteristics since if the features are irrelevant, our outcome will be ambiguous, incorrect, and noisy, potentially affecting decision-making. If the feature is relevant, on the other hand, it allows us to assess and make decisions based on more dimensions that were previously disregarded. Volume is a distinct feature that does not overlap with established business model elements. By adding “Volume” with the existing business models, not only the segments with potential customers have been recognized but also the negative profit driving segments have been identified which can be seen in Fig 13. Furthermore, the segment numbers for the new LRFMV model are higher than for previous models, allowing us to clearly identify distinct sorts of consumer segments with successful business solutions which is visible in Table 7.

From the dataset, the features which solely focus on profit maximization have been considered. The higher the number of purchased products by the customer, the higher the chance of maximizing profit. Therefore, there is a direct relationship with the profit maximization and purchase quantity in any business corporations. We normalized the number of products purchased by a potential consumer over a certain length of time or a set number of visits to determine a group of valuable customers for superstores. When popular existing RFM and LRFM models are compared to the LRFMV model in section 4.1, more categories and new enterprise insights can be generated, and the LRFMV model beats the prior ones. Three clustering strategies were applied for improved clarity, and the segments of LRFMV were able to produce more significant results in each technique. Following that, it is revealed that the higher the increase in volumes, the higher the profit for the majority of the segments which can be seen in section 4.2. Therefore, there exists a direct relationship between profit and volume in the case of superstores which can be a potential key to implement some effective business solutions. As a result an appropriate customer segmentation can be achieved using different clustering algorithms and further analyzed using classification matrices.

Reviewer#2, Concern # 3: More exhaustive evaluation would be helpful.

Author response: Thank you for your valuable suggestions. As per the respected reviewer’s recommendation, we have appended a customer profitability matrix for better inception of the research.

Author action: Updated sections have been marked yellow using the highlighter tool in the revised

manuscript.

Modifications made to the manuscript in this regard is as follows:

[Updated] [Section: 4.2 Result Analysis]

The goal of this paper is to bring business and technology together to boost profits for superstores. Customer profitability measurement is critical for long-term business performance since it allows us to observe if particular clients are costing us money rather than making money. It becomes simple to examine client profitability as much as an organization requires once it has a structure in place to measure it. A consumer segment can be proved less important to the business than others which were previously considered profitable before analyzing them by using any metrics.

These results from different customer profitability metrics can then be used to develop and shift business strategies in order to maintain business objectives and goals on track. As an enhanced business model LRFMV, is proposed in this research to locate successful customer segments for the superstore business, it is necessary to evaluate customer segments which have been created by using different clustering algorithms by using various business metrics to offer organization management with an awareness of each customer profitability. Among different customer Customer Profitability Analysis, strategy alignment and customer profitability matrix is one of the most important measuring tools for grouping information into customer profitability segments which allows companies to take different, targeted actions and strategies against different profitability segments with the goal of increasing the company's total profitability.

In Table 8, all consumers are classified into four groups, each with its own method for dealing with customers

Table 8. Analysis of profit for each cluster and identifying potential customer segment using Customer Profitability matrix

Retain<br>(Profitable, Target)	Transform<br>(Unprofitable, Target)
Monitor<br>(Profitable, Non-target)	Replace<br>(Unprofitable, Non-target)

Profitable clients in the **"Target"** group are linked to the action **"RETAIN"** and the company should investigate the prospect of expanding commercial connections with such customers, as long as the business model does not change considerably.

Profitable customers in the **"Non-target"** group are linked to the act **"MONITOR"** and these consumers must be regularly watched to avoid falling into the **"Non-target"** and unprofitable sector.

Unprofitable customers in the **"Target"** group as related to the action **"TRANSFORM"** and the corporation should use various techniques to convert these customers into profitable segments, or at the very least, bring them to a cut off point. Depending on the company-customer business circumstances, different tactics will be employed.

Unprofitable clients in the **"Non-target"** category are linked to the action **"REPLACE"** to whom the company should stop investing in their development. The suggested answer is to raise product or service selling prices until the consumers fall into the **"MONITOR"** sector or move their business to another provider. If this occurs, the corporation may be able to refocus its efforts toward serving the most profitable customers.

If we utilize strategy alignment and customer profitability matrix to evaluate our findings of 5 separate clusters of customers via centroid-based algorithms, we will get the following results. Out of the five client clusters, the first four are lucrative, while the fifth, cluster 4, is non-profitable with a negative value of -262.818. Cluster 1 is the most profitable client segment, with a total profit of 1371432. Clusters 1 and 3 are considered successful since they generate a profit greater than their average profit, as seen in Table 9.

Table 9. Customer Type Analysis and Target Audience Identification for each cluster

Cluster	Profit	Target Audience	Customer Type
**Cluster 0**	17645.83	No	Monitor
**Cluster 1**	1371432	Yes	Retain
**Cluster 2**	68997.14	No	Monitor
**Cluster 3**	51603.23	Yes	Retain
**Cluster 4**	-262.818	No	Replace

This matrix boosts profits by removing unprofitable consumers and increasing sales or services to profitable ones. It is an assessment of the true costs of each client group, including taking non-production expenses into account when calculating profitability, and it indicates that non-production costs can occasionally surpass production costs.

Reviewer#2, Concern # 4: Legend for Fig. 5 needs to be updated. Currently this figure is meaningless without proper bar labels.

Author response: We would like to thank the honorable reviewer for the intuitive comment and suggestion. We have incorporated your recommendation in the revised version of this manuscript. For better explanation, instead of a bar chart we have included the values of two-dimensional principal components for the LRFMV model with scatter plots in Figure 5. Besides, we have also added a depiction of K-Means clusters over PCA components for the LRFMV model. To facilitate visualization, the clusters are labeled with six distinct hues.

Author action: Modifications made to the manuscript in this regard is as follows:

[Updated] [Section: 3.4.3 PCA]

In Fig 5, it is visible that information of 5 clusters of the LRFMV model has been represented in two dimensions by using PCA to avoid the convolution.

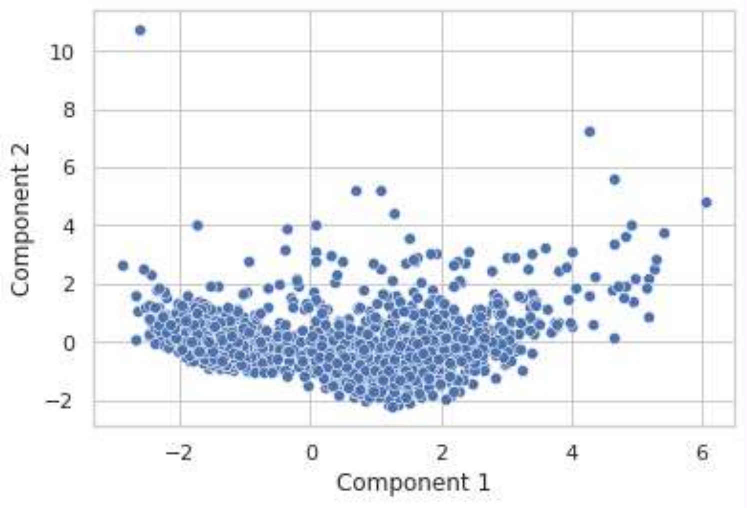

Figure 5. PCA on the proposed dataset

In Fig 6, the K-Means clustering algorithm has been applied over PCA components for the LRFMV model. It is visible that 5 clusters have been formed after applying the K-Means algorithm which has been represented by 5 distinct colors.

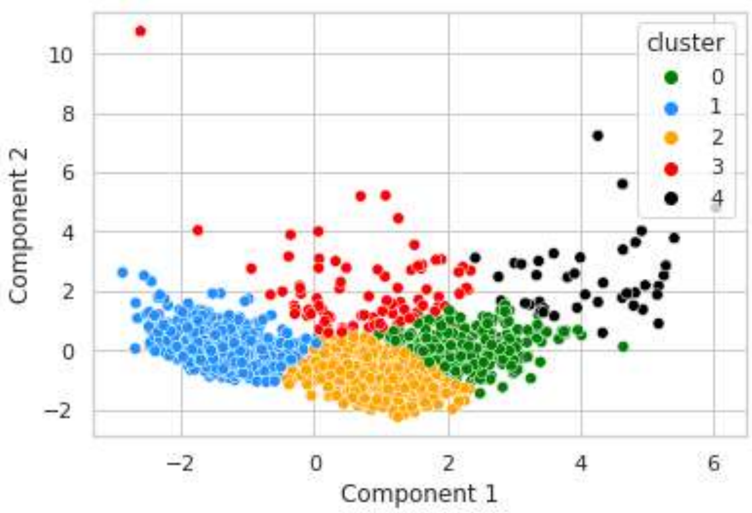

Figure 6. K-means clusters over PCA components for LRFMV model

JOURNAL REQUIREMENTS

Concern # 1: Clarify in your Methods section whether the customer names and other identifying characteristics in the dataset used are fictional.

Author response: Tableau software is recognized for dealing with real-world information and doing analysis to give prospective results to its clients; yet, due to client confidentiality, they are unable to release any datasets directly. Tableau data sets are collections of data that come from a variety of sources and are used to visualize data. Here, personal identifiable information is always treated with confidentiality. As a result, our concern dataset blends real-world facts with fictional personal information. The dataset we used in the research is collected from "data.world" which is a customer-centric data collection that includes information on all orders placed through various suppliers and markets from 2011 to 2014. As recommended, we have clarified the issue regarding the dataset in the revised manuscript.

Author action: Updated sections have been marked yellow using the highlighter tool in the revised

manuscript.

Modifications made to the manuscript in this regard is as follows:

[Updated] [Section 3.2 Dataset Description and Data Preprocessing]

Superstore business has been a growing and profitable business for the last few decades worldwide, and the process of finding potential and profitable customers will be beneficial for this sector. This research aimed to scrutinize the cluster of potential buyers by preprocessing and analyzing a standard superstore dataset. This research used the global superstore dataset [38] of Tableau community that is a publicly available open dataset. The Global Superstore data set has around 50000 records, and it is a customer-driven informational dataset that contains comparative data of orders placed through various sellers and markets from 2011 to 2014. Customer order shipment date, city, category, and order priority are among the 24 attributes included in the dataset. The presented customer names and addresses in the dataset are fictional.
