# Supplementary material for: LRFMV: An efficient customer segmentation model for superstores
Source: PLoS One. 2022 Dec 20;17(12):e0279262. doi: 10.1371/journal.pone.0279262 (PMC9767363; doi:10.1371/journal.pone.0279262)
Supplement: Attachment — Submitted filename: Response to Reviewers.pdf [file pone.0279262.s002.pdf]

Original Manuscript ID: PONE-D-21-32764R1

Original Article Title: “LRFMV: An Efficient Customer Segmentation Model for Superstores”

To: The Editor in Chief, PLOS ONE

Re: Response to reviewers

Dear Sir,

We appreciate you allowing us to resubmit our article so that we can address the reviewers' recommendations. We want to offer our sincere gratitude and appreciation for the comments, which really helped us to improve our manuscript. The manuscript was rewritten after carefully examining all the comments. When responding to a comment, we first provide a general summary of the issue before outlining the key adjustments made to the revised document. Below are detailed responses and justifications to the reviewers' comments and recommendations.

We have uploaded (a) a point-by-point response to the reviewers' suggestions (answer to reviewers), (b) an updated manuscript with yellow highlighting showing changes, and (c) a clean, updated version without highlights (reaction to reviewers) (PDF main document).

In the response letter, we have responded to the comments of Reviewer 1 and Reviewer 3 respectively.

Thank you for your consideration.

Best regards,

Dr. Md. Golam Rabiul Alam

(Corresponding Author)

RESPONSE TO REVIEWER #1'S COMMENTS

Reviewer#1, Comment: Authors have addressed all my comments in the revised manuscript. I don't have any further comments on this manuscript.

Author response: We want to express our sincerest appreciation to the respected reviewer for the reviews and feedback given in the previous round of review. Your comments and reviews surely help us to improve the quality of our research work and the manuscript as well. Thank you for your positive comment on our research work.

RESPONSE TO REVIEWER #3'S COMMENTS

Reviewer#3, Concern # 1: With the features of the classical LRFM model, it is possible to identify customer groups, such as profitable, non-profitable, potential, uncertain, loyal, new, and etc. Thus this model is utilized by many researchers in different application domains to analyze customer behaviors, to identify different and target customer groups. Considering these, how will this new feature, the purchased quantity contribute to the modelling of customer behaviour? Are there any studies showing the effect of this feature on modelling customer behavior or customer profitability? Is there any study showing the direct relationship between profit and quantity? The study lacks literature supporting the profit-amount relationship on which the proposed model is based.

Author response: We want to express our sincere appreciation to the respected reviewer for his thoughtful feedback. In the Literature Review section, we have included some studies based on the profit-quantity relationship. Moreover, in the result analysis section of the revised article, we have added a paragraph discussing the profit-volume relationship of this research as suggested by the esteemed reviewer.

In this research article, we suggest a term that enhances the LRFM model by identifying the customer clusters that increase an organization's profitability. It demonstrates that regardless of the amount of money spent, a consumer will contribute more to the organization's profit if he purchases a significant amount of goods throughout specific trips. The possibility of maximum profit increases with the number of products a customer purchases. The following part, which can be found in section 4.2 known as result analysis, reveals that the majority of the sectors' profits increase in direct proportion to sales volume. In the case of superstores, there is a clear correlation between profit and volume, which may be key to implementing some efficient business strategies. We have shown the profit-volume relationship in bar charts for the three clustering methods utilized in this paper, K-Medoids, Mini Batch K-means, and K-means, in figures 11 to 13.

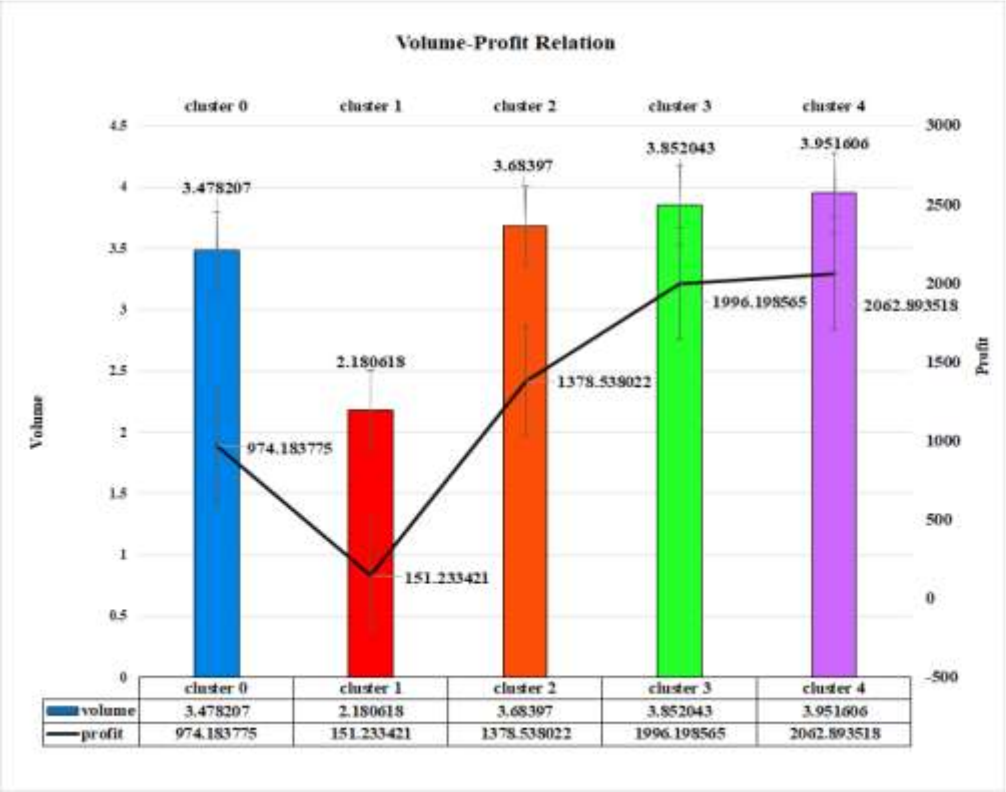

Figure 11. Volume-Profit Relationship of LRFMV model for K-Medoids Algorithm

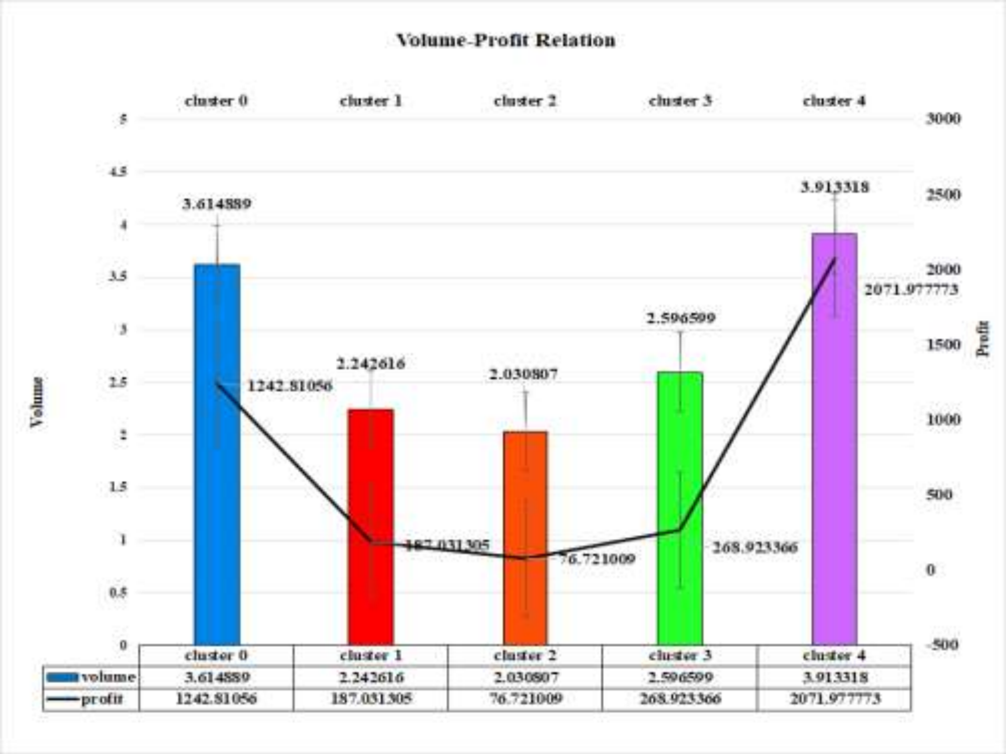

Figure 12. Volume-Profit Relationship of LRFMV model for Mini Batch K-Means Algorithm

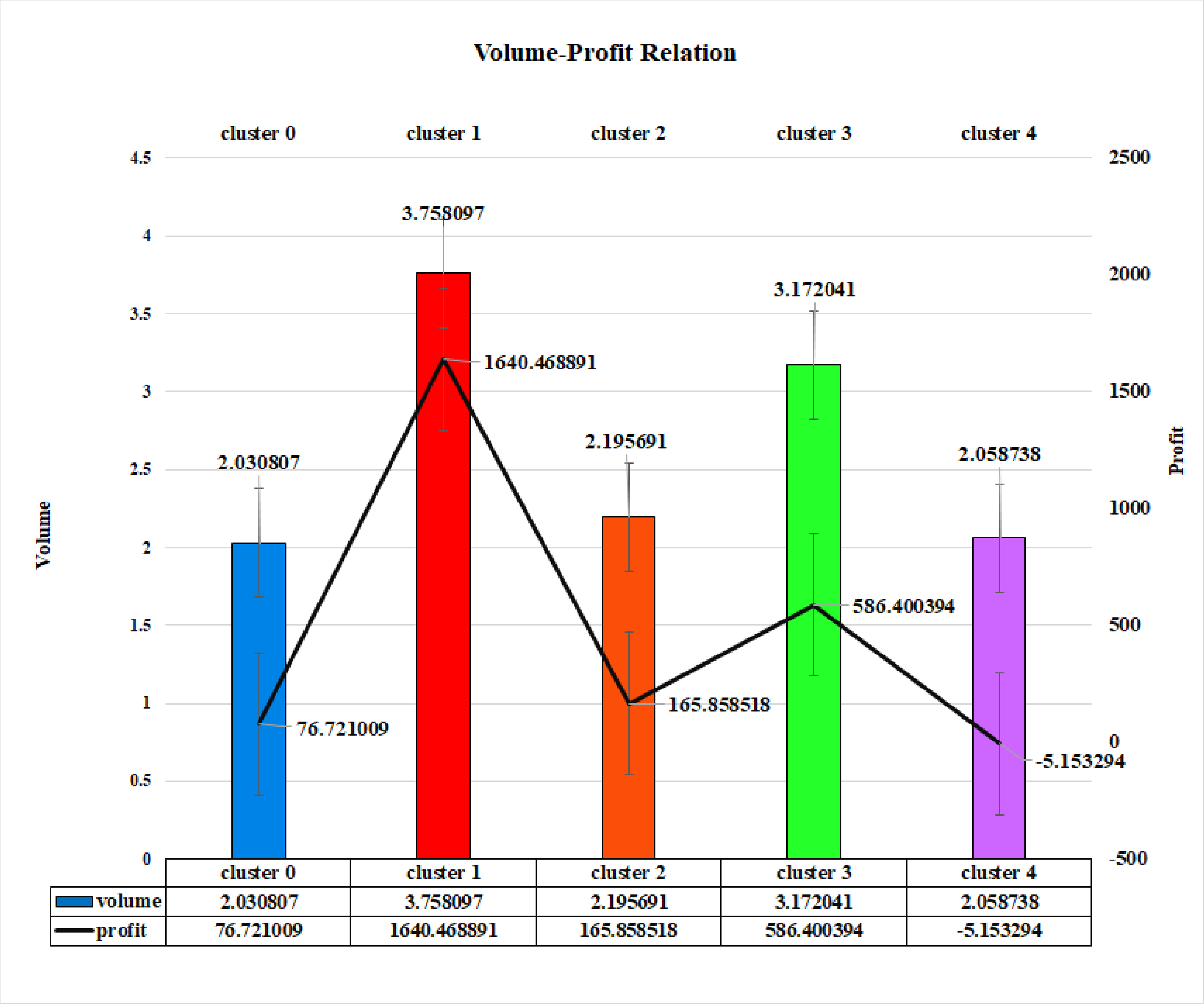

Figure 13. Volume-Profit Relationship of LRFMV model for K-Means Algorithm

Author action: Updated sections have been marked yellow using the highlighter tool in the revised manuscript.

Modifications made to the manuscript in this regard is as follows:

[Updated] [Section: 2 Literature Review]

Over the years, many data mining techniques have been widely applied in a variety of fields. As the volume of transactions in the business grows, it has become more difficult to segment profitable customers to enhance sales. "Quantity" has been incorporated in different studies for profit maximization as well as association rule mining [44] [45]. The volume or quantity produced directly relates to the revenue, cost, and profit function[2]. Lau. J. [42] explored several applications of profit function through an alternate derivation to characterize production and profit function. According to the study, the firm maximizes profit with the optimized quantities of the variable inputs where maximization of the profit is similar to the

normalized profit function. Chen. Chung-Ho [43] et al. presented the supply chain system's optimal profit-sharing model between producers and consumers. According to the study, the order quantity contributes to the average consumer demand and significantly impacts the predicted profits of the supplier and the buyer.

[Updated] [Section: 4.2 Result Analysis]

To establish and explore new business opportunities in today's world, it has become essential to mix technical innovation and knowledge with business. In light of this, the goal of this article was to provide a novel technology element that would enable an organization to increase its profitability. In order to find the consumer group that would guarantee and provide a larger profit to a business, a new feature V has been introduced to the existing LRFM model. It can be noticed clearly that the profit-volume relationship is proportionate, as shown by the preceding figures (figure 11-13), and any business can generate a sizable profit by analyzing the volume, which is a scaled-down representation of the quantity of things purchased by a potential consumer. By emphasizing the amount of product those clients have purchased over a predetermined time frame or a predetermined number of visits, it helps any business identify a group of valuable customers.

Reviewer#3, Concern # 2: Moreover, the authors provide Fig 7 to represent the relationship between each parameter in the model. Authors assert that the newly introduced parameter volume (V) has a very weak relationship with each of the other parameters and is quite strong with itself. Thus, they conclude that V is a unique feature and can not be replaced by other features. However, when we observe that figure, it is clearly seen that the new proposed feature volume (V) directly correlates with both frequency (around 0.8) and monetary (around 0.6). These values are quite high for the correlation analysis. When this is taken into account, it is seen that the new feature does not add any extra dimension to the current model.

Author response: We want to express our sincerest appreciation to the respected reviewer for raising the interesting fact that the newly proposed feature volume (V) directly correlates with both frequency (around 0.8) and monetary (around 0.6). In the customer segmentation problem, it is necessary to find all the relevant factors to group similar customers together. In contrast, in the feature selection problem, we need to eliminate features with high correlation to remove the redundant features. Therefore, even in the existing LRF and LRFM model, the factor length (L) and frequency (F) exist together although these two factors have a higher correlation (around 0.8) [Please see Figure. 7]. The correlation between frequency (F) and monetary (M) is also high (around 0.5).

We proposed to add volume as a new component to the LRFM model with the goal of presenting a comprehensive and appropriate solution for evaluating various datasets. No feature can be said to be indispensable at first, but based on its application to our real-world dataset and the findings, we can say that it will undoubtedly play a key role in conjunction with the current LRFM model. However, we exercised caution while choosing characteristics since if a feature is no longer relevant, the results would be unclear, inaccurate, and noisy, which may have an impact on decision-making. Our goal of advancing business analysis and profit maximization will be successful if a new potential feature can be included because it will enable us to evaluate and make judgments based on more factors that were previously overlooked. By

incorporating "Volume" into the current business models, not only were the sectors with potential customers recognized, but also the segments that drove down profits, as shown in Fig. 13. Additionally, the new LRFMV model's segment numbers are higher than those of earlier models, enabling us to easily distinguish various consumer segment types that have profitable business solutions, as shown in Table 7. After considering these effects of volume, it can be considered as a potential feature in spite of having a correlation with frequency and monetary.

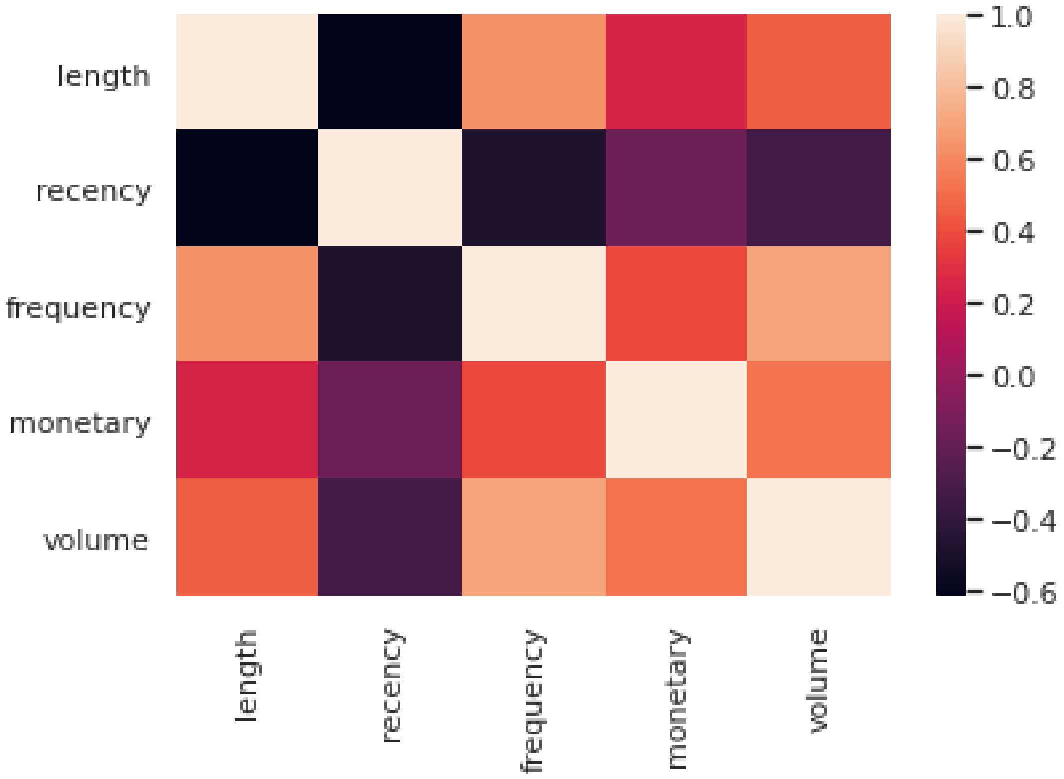

Figure 7. Heatmap for LRFMV model

Author action: Updated sections have been marked yellow using the highlighter tool in the revised

Manuscript.

Following are the changes made to the manuscript in this regard:

[Updated] [Section: 3.5 Correlation of volume with other features]

As an upgraded version of the RFM model, the Length, Recency, Frequency, and Monetary model (also known as the LRFM model) was developed to identify more precise and pertinent consumer groups for profit maximization. The properties of the current LRFM model, as seen in Figure 7, likewise demonstrate a good correlation between length and frequency (0.8) and between monetary and frequency (0.6). Even yet, many researchers utilize this strategy frequently to successfully analyze client behavior across a range of application fields and in spite of having a good correlation with frequency, length has been introduced.

So we believe that volume can help to further assess how much revenue boost and marketing strategy can be developed for the superstore industry and contribute to both technical sectors and the business world.

Reviewer#3, Concern # 3: Authors state that the volume highlights the customers who provide more profit to the organization. However, customer profitability is not related to the amount purchased. Monetary (M) and frequency (F) variables explain customer profitability in the standard model. Let's try to explain with an example that the amount purchased does not add an extra dimension and is a meaningless variable in terms of customer segmentation. There is a customer who buys a lot of products, but that customer may only buy certain products from the relevant supermarket that are quite cheap. In other words, the total amount paid is quite less. In this case, when we consider the amount received, how can we call this customer profitable? Or let's think the opposite, let's say that another customer who buys very few products, and the products he buys are quite expensive. In other words, the total amount paid is quite high. In this case, when we consider the amount received, how can we call this customer non-profitable? Therefore, purchased quantity as seen in the example, does not offer an extra dimension in explaining customer behaviour.

Author response: Superstores carry a wide variety of goods and some have a history of being purchased by the same client multiple times on the same day in various quantities. The relationship between profit and purchased quantity and how they can support an efficient consumer behavioral analysis were not looked into and assessed in the RFM and LRFM models. As superstore customers were our target group, it can be said that superstores don't stock particularly expensive items, and most customers choose to buy necessities from superstores rather than luxury goods. There are some expensive items but the customers do not buy them frequently which is the same for the cheaper items. According to the equation of volume, the number of days while transacting in the customer's life cycle is also measured. Again, the price of the common items frequently bought by the customers does not vary much in the case of the superstores which is different for the electronic shops. We have also included references for this which includes the most common items bought in a superstore and these are mostly items needed for daily groceries. In light of this, we suggested a feature that will enhance their business analysis and, indirectly, assist them in determining their best-selling products for boosting sales. To do so, we relied on our real-world dataset instead of case studies.

Author action: Updated sections have been marked yellow using the highlighter tool in the revised

Manuscript.

The following changes have been made to the manuscript in this regard:

[Updated] [Section: 4.1 Comparative Analysis]

From comparative analysis, we can see that the LRFMV model is providing a reasonable output compared to the RFM and LRFM models and is ensuring the polarity, which is not present in RFM or LRFM models for any of the three clustering algorithms. We suggested volume, which was determined by assessing the typical number of items a regular superstore customer purchases in a single day. The previously discovered average quantity was divided by the entire number of days in the customer's brief period of visitation in

order to obtain the final average as the volume in a given time frame. There are some expensive products in the superstores as well as very cheap products. However, the price of the most common items frequently bought by the customers are usually daily groceries and do not vary much[3,4,5]. Additionally, the LRFMV model has one additional cluster while using the K-Medoids algorithm, to increase profitability and the overall profit margin is higher than other models, where both RFM and LRFM models do not have the additional cluster, which also guarantees superior clustering quality. In the bar chart of figure 9, which compares the profitability of three distinct models, it can be seen that the LRFMV model's cluster which is represented by the green bars makes the most profits for clusters 0, 1, and 2 compared to LRFM and RFM models for Mini batch K-Means Algorithm. Furthermore, while the LRFMV model uses K-Means Algorithm to produce a structured output, RFM and LRFM models reveal conflicting profit analyses for various clusters. We can assert that this has occurred in order to add volume to the existing models because the LRFMV model is producing a passable output when compared to the RFM and LRFMV models.
